# A Complete Optical Sensor System Based on a POF-SPR Platform and a Thermo-Stabilized Flow Cell for Biochemical Applications

**DOI:** 10.3390/s16020196

**Published:** 2016-02-05

**Authors:** Nunzio Cennamo, Francesco Chiavaioli, Cosimo Trono, Sara Tombelli, Ambra Giannetti, Francesco Baldini, Luigi Zeni

**Affiliations:** 1 Department of Industrial and Information Engineering, Second University of Naples, Via Roma 29, Aversa 81031, Italy; luigi.zeni@unina2.it; 2 Institute of Applied Physics “Nello Carrara”, CNR, Via Madonna del Piano 10, Sesto Fiorentino 50019, Italy; f.chiavaioli@ifac.cnr.it (F.C.); s.tombelli@ifac.cnr.it (S.T.); a.giannetti@ifac.cnr.it (A.G.); f.baldini@ifac.cnr.it (F.B.)

**Keywords:** flow cell, surface plasmon resonance, plastic optical fiber, sensors system, biosensors

## Abstract

An optical sensor platform based on surface plasmon resonance (SPR) in a plastic optical fiber (POF) integrated into a thermo-stabilized flow cell for biochemical sensing applications is proposed. This device has been realized and experimentally tested by using a classic receptor-analyte assay. For this purpose, the gold surface of the POF was chemically modified through the formation of a self-assembling monolayer. The surface robustness of the POF-SPR platform has been tested for the first time thanks to the flow cell. The experimental results show that the proposed device can be successfully used for label-free biochemical sensing. The final goal of this work is to achieve a complete, small-size, simple to use and low cost optical sensor system. The whole system with the flow cell and the optical sensor are extensively described, together with the experimental results obtained with an immunoglobulin G (IgG)/anti-IgG assay.

## 1. Introduction

A fast and reliable detection of analytes (such as proteins, viruses, bacteria, toxins, DNA and chemicals in general) at very small concentration is essential in many fields, ranging from medical diagnostics, pharmaceutical research, environmental monitoring and security to quality control in the food industry. One of the most used approaches to detect a chemical/biochemical interaction taking place on a functionalized surface is based on the measurement of the related refractive index (RI) change [1]. In this context, optical sensing platforms, such as surface plasmon resonance (SPR) [2], localized SPR (LSPR), interferometric configurations [3], resonating structures and optical fiber gratings [4,5], represent valid and widespread options with respect to electronic and silicon-based devices.

SPR-based biochemical sensors using optical fibers have been shown to be able to play an important role in RI sensing, especially where fast, portable, low cost and rugged units are needed for early detection and identification [6,7,8,9,10,11]. Among the existing device configurations, those based on fiber tips along with the use of nanoparticles or other nanomaterials are one of the most investigated [12]. These small-scale objects allow a wide range of possibilities to modify the device in the sensitive area, enabling also unique effects such as LSPR. Another mostly-studied configuration involves the use of fiber tapers [13]. Nowadays this technology has reached enough maturity to be a good choice for any kind of chemical and biological SPR-based sensor. In any event, the performance of the existing SPR-based optical fiber sensors generally depends on the adhesion of the metallic layer (commonly gold) to the fiber surface [14]. SPR is known to be a very sensitive technique for determining RI variations at the interface between a metallic layer and a dielectric medium (buffer solution or real fluid containing the analyte under investigation). In practical implementations, the biological targets are usually transported through a microfluidic system [15] by means of a buffer solution or a carrier fluid. In general, the optical fiber employed can be either a glass optical fiber or a plastic one.

For low-cost SPR-based sensing systems, plastic optical fibers (POFs) are especially advantageous due to their excellent flexibility, easy manipulation, great numerical aperture, large diameter, and the fact that plastic is able to withstand smaller bend radii than glass. The same optical sensor platform based on SPR in a POF has already been tested in different biochemical applications and in low molecular weight substance detection [16,17,18], using different receptors, without the integration into a thermo-stabilized flow cell system.

Here, the first example of the POF-SPR biosensor with a flow cell system for selective detection of a specific biological target (analyte) is presented. In this work, IgG/anti-IgG assay was implemented as exemplifying bioassay, with the IgG biolayer deposited on the gold surface and the biological target, anti-IgG, transported through a new thermo-stabilized flow cell by means of a buffer fluid. As a preliminary feasibility test, human serum with IgG spiked in was used to mimic a more real and effective measuring environment. This complete optical sensor system can be used for the future reduction of the device cost and dimension, with the possibility of integrating the POF-SPR sensing platform with microfluidic and optoelectronic devices, eventually leading to a “lab-on-a-chip” device [15,19].

## 2. Experimental Section

### 2.1. Materials

Ethanol (EtOH), 11-mercaptoundecanoic acid, bovine serum albumin (BSA) and all the reagents for buffer preparation (phosphate-buffered saline (PBS), 40 mmol/L, pH 7.4) were purchased from Sigma-Aldrich (Milan, Italy). Mouse IgG and goat anti-mouse-IgG were purchased from Zymed Laboratories, Invitrogen Immunodetection (Milan, Italy). 1-Ethyl-3-[3-dimethylaminopropyl] carbodiimide hydrochloride (EDC) and N-hydroxysuccinimide (NHS) were purchased from EuroClone (Milano, Italy). Human serum (C Reactive Protein Free Serum) was purchased from HyTest Ltd. (Turku, Finland). Polydimethylsiloxane (PDMS, Sylgard® 184 silicone elastomer kit) was purchased from Dow Corning (Wiesbaden, Germany).

### 2.2. Optical Sensor Platformand Experimental Setup

**Figure 1:**
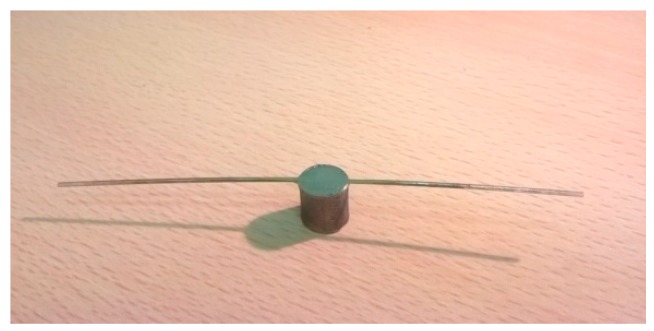
Picture of the POF-SPR sensing platform.

A picture of the POF-SPR platform is shown in Figure 1. The plastic optical fiber has a PMMA core of 980 μm and a fluorinated polymer cladding of 20 μm. The previously-reported experimental results indicate that this configuration with a fiber of 1000 μm in diameter exhibits good performances in terms of RI sensitivity and resolution but it is less performing in terms of signal to noise ratio (SNR) with respect to the same platform exploiting smaller diameter of the fiber [20,21,22]. However, as stated in our previous work [16], the choice of a multimode plastic fiber having a larger core diameter, derives from the need to realize an easy-to-handle and low-cost device. The fabricated optical sensor platform was realized removing the cladding of the fiber along half the circumference, spin coating on the exposed core a buffer of Microposit S1813 photoresist (MicroChem Corp, Westborough, MA, USA) with a thickness of about 1.5 μm achieved with a spin speed of 3000 rpm, and, finally, sputtering a thin film (60 nm) of gold using a SCD 500 sputtering machine (Leica Microsystems, Wetzlar, Germany) ensuring its uniformity and repeatability, as described in previous literature [20,23].

The photoresist buffer layer between the gold film and the POF core is necessary to obtain an enhancement of the plasmonic resonance, which is due to the presence of the high refractive index photoresist layer [23]. Actually, in the visible range of interest, the refractive index is about 1.49 for PMMA (POF core), 1.41 for fluorinated polymer (POF cladding) and 1.61 for the Microposit S1813 photoresist buffer layer.

The realized sensing region was about 10 mm in length. In order to obtain a good robustness of the gold film it is necessary to sputter the gold film after having waited at least one day after the spinning of Microposit S1813 photoresist, which is an electronic-grade product ensuring a good adhesion of the metal film to its surface, after the complete evaporation of the solvent (propylene glycol monomethyl ether acetate [24]).

In this paper, the robustness of the gold film has been tested by implementing a bioassay under flow conditions, as it will be presented in the next sections. The experimental setup was arranged to measure the light spectrum transmitted through the POF and was characterized by a halogen lamp as optical source and by a spectrum analyzer [20,23]. The employed halogen lamp exhibits a wavelength emission range from 360 nm to 1700 nm, while the detection range of the spectrum analyzer (USB2000+VIS-NIR spectrometer, Ocean Optics, Dunedin, FL, USA) was from about 330 nm to 1100 nm. The spectrometer is finally connected to a computer for the data processing and analysis. The SPR curves along with the minimum of the SPR resonance wavelength were displayed online on the computer screen and saved with the help of advanced software provided by Ocean Optics. The processing of the measured data was carried out by Matlab software. The SPR transmission spectra were obtained by measuring the transmission spectra normalized to the spectrum achieved with air as the surrounding medium.

### 2.3. Flow Cell System

**Figure 2:**
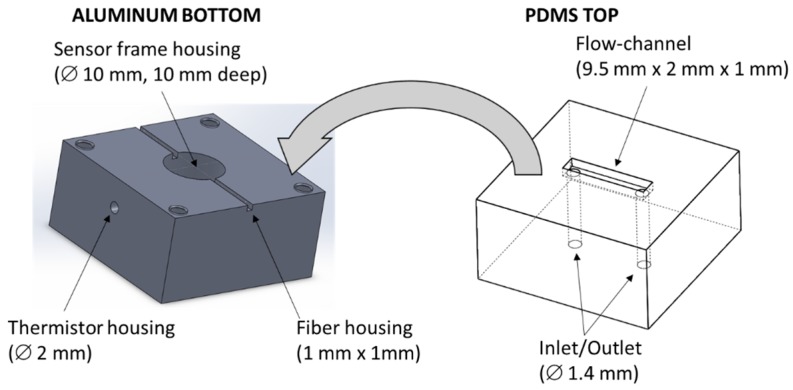
On the left: The flow cell aluminum bottom part with the sensor frame housing for the plastic optical fiber, and the thermistor housing. On the right: The PDMS top part, with the flow-channel (9.5 mm long, 2 mm wide, 1 mm deep) and the holes for the fluid inlet and outlet.

The flow-cell is schematically depicted in Figure 2. It is composed of two parts: (1) the aluminum bottom part containing the housing of the sensor frame, the groove housing of the plastic optical fiber and the hole housing of the thermistor.

The dimensions of the bottom part are: 30 mm × 30 mm × 15 mm. (2) The PDMS top part with the flow-channel and the inlet and outlet. The dimensions of the flow channel are: 9.5 mm × 2 mm × 1 mm and the volume is about 20 µL. The total dimensions of the PDMS part are: 20 mm × 20 mm × 10 mm. The optical fiber sensor, mounted on its plastic frame (see Figure 1), fits perfectly into the sensor frame housing obtained into the aluminum bottom part so that, when the PDMS part is placed on the top, the flow-channel fits only in the area where the sensing region of the optical fiber.

**Figure 3:**
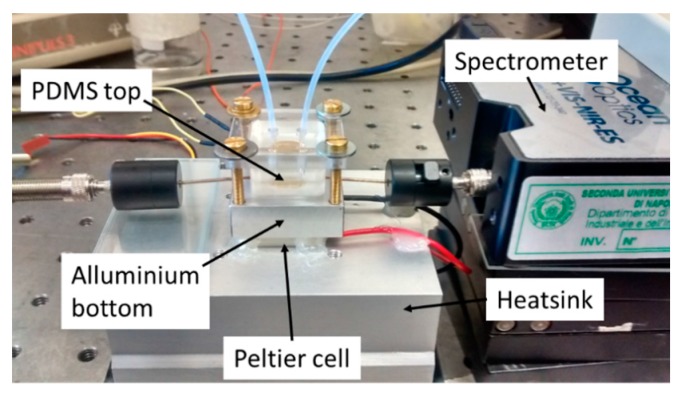
Picture of the experimental setup.

A picture of the experimental setup with the thermo-stabilized flow cell system is shown in Figure 3. The aluminum bottom part of the flow cell is mounted in thermal contact with a Peltier cell (20 mm × 20 mm) and a thermistor is inserted into the lateral hole (see Figure 2). A Thermo-Electric-Cooling (TEC) driver (ILX LIGHTWAVE LDC-3722B, Bozeman, MT, USA) acquires the temperature from the thermistor and this value is used as feedback to drive current to the Peltier cell in order to lock the temperature at 23 °C (±0.1 °C). The Peltier cell, in its bottom part, is in contact with a heat-sink to increase its thermal efficiency. The PDMS top part is stuck to the bottom part and to the sensor cylindrical plastic frame by means of a PMMA slide and four screws. In this way, thanks to the mechanical characteristics of the PDMS, it is possible to completely seal the flow channel. The transparency of the top part allows checking the filling of the flow channel and, eventually, the formation of air bubbles.

### 2.4. Sensor Functionalization

The functionalization of the sensor gold layer was achieved by the deposition of 11-mercaptoundecanoic acid 0.5 mmol/L in a H_2_O/ethanol solution (10% ethanol). The thiol solution was left in contact with the gold layer for 12 h. Once the sensor surface was functionalized, the fiber was placed inside the thermo-stabilized microfluidic system. All the steps for the implementation of the immunoassay were performed using the flow cell connected to a peristaltic pump and keeping the temperature of the flow cell at (23 ± 0.1) °C. The preparation of the biolayer (receptor) consisted of the following steps: activation of –COOH (carboxylic) groups by cross-linking chemistry (200 mmol/L EDC and 50 mmol/L NHS), covalent immobilization of mouse IgG (1000 mg/L in PBS), washing with PBS for removing the un-reacted antibodies, and surface passivation with BSA (1% in PBS) in order to block the remaining activated carboxylic groups and to prevent non-specific adsorption onto the surface.

### 2.5. Protocol of the Measurements in Buffer and Human Serum

In this work, we have used a standard protocol to always measure the changes in refractive index due to the bio-interaction under the same conditions. In particular, a washing step with PBS (buffer) after the bio-interaction was carried out and the spectrum was recorded after stopping the flow thus with stable thermal and hydrodynamic conditions. Although the measurement under flow conditions could be used to exploit the biosensor interactions, it should be pointed out that the important measurand is constituted by the RI changes induced along the functionalized surface due to the specific interaction between the immobilized antibody and the antigen, and not by bulk RI changes, simply related to the refractive index of the solutions which are pumped inside the flow cell. This means that a washing step with PBS is necessary after each addition of the sample in order to measure the real shift of the resonance determined by the antibody/antigen interaction on the fiber surface.

The antigen binding steps, with increasing concentrations of goat anti-mouse IgG ranging from 10 µg/L up to 500 mg/L in PBS, were performed by flowing the antigen solution onto the modified sensor for 15 min followed by a washing step with PBS (buffer) of 5 min. The same assay was also performed by spiking human serum (diluted 1:10 (*V/V*) in PBS) with goat anti-mouse IgG at increasing concentrations (from 10 µg/L up to 10 mg/L). Also in this case, the measurement of the wavelength shift has been performed after the washing of the sensor by means of PBS buffer, in order to detect the RI change induced only by the bound analyte, without any possible perturbation induced by bulk refractive index differences.

## 3. Results and Discussion

The feasibility and integration of the POF-SPR sensor platform with the *ad hoc* developed microfluidic system were evaluated performing the model IgG/anti-IgG assay both in buffer solution (PBS) and under more realistic and effective conditions, *i.e.*, in human serum. Two different plasmonic resonances were observed in the considered wavelength range (300–1100 nm) of the transmission spectra [25], when the binding interaction occurs between bio-receptor and analyte under investigation. In particular, the resonance displaying a blue-shift was at shorter wavelengths with respect to the other one that displayed a red-shift of the SPR resonance [25]. Since the minimum at shorter wavelengths is much better defined than the other and the blue-shifted resonance is characterized by a greater response to the analyte concentrations, only this resonance will be taken into account hereafter. Two POF-SPR sensors were used to carry out the two experiments in buffer and in serum. The two sensors exhibited the main SPR resonance placed at two different starting wavelengths (even if the device response showed the same trend): 576 nm for the device used in buffer, whereas 626 nm for that one used in serum.

### 3.1. Assay in Buffer

**Figure 4:**
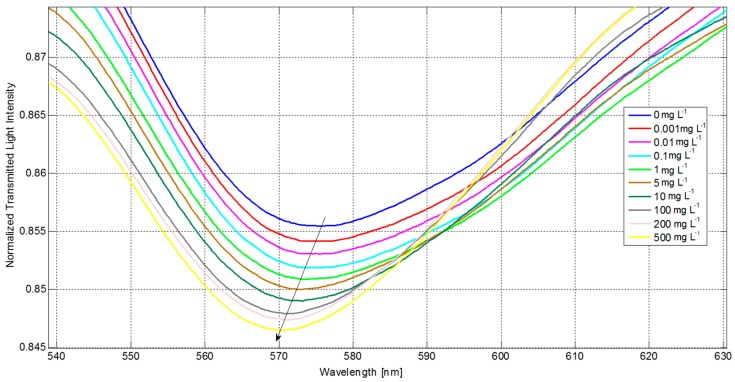
Normalized SPR spectra acquired by the POF-SPR biosensor for different analyte concentrations in buffer.

Figure 4 shows the shift of the SPR resonance at around 576 nm, obtained for different concentrations of the analyte (from 0 to 500 mg/L) in buffer solution: the resonance wavelength is shifted to lower values when the analyte concentration increases, indicating that the analyte effectively interacts with the immobilized receptors from the buffer matrix here considered. The increase in depth of the resonance peak exhibiting the blue-shift is a direct consequence of the stronger excitation of the surface plasmon, which depends on the effective RI of the layer.

**Figure 5:**
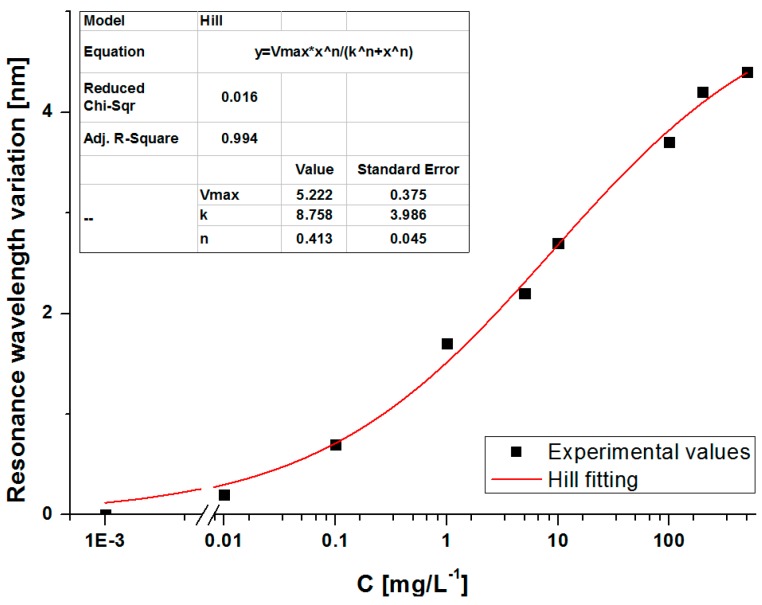
Absolute values of resonance wavelength shift versus the concentration of analyte and the fitting of the data by Hill equation. Inset: Hill parameters.

Since the parameter of interest is the measurement of the resonance wavelength shift, any changes in the resonance depth do not affect the results, as far as the peak remains clearly distinguishable. By extracting the minimum of the SPR resonance for each analyte concentration, it is possible to draw the calibration curve of the sensor. The absolute value of the wavelength shift of the transmission minimum (Δλ) versus the concentration of the analyte is shown in Figure 5, together with the Hill fitting of experimental values [18,25]. The results depicted in Figure 5, demonstrate that the system is able to detect concentrations of the analyte under investigation (IgG) lower than 0.01 mg/L.

### 3.2. Assay in Human Serum

In order to prove that the new complete optical sensor system can be exploited in real biochemical applications, which require a thermo-stabilized microfluidic system, the same IgG/anti-IgG assay was performed under more realistic and effective conditions, *i.e.*, in serum.

**Figure 6:**
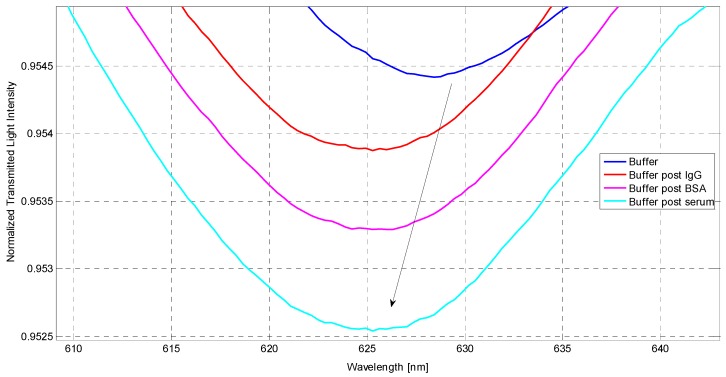
Normalized SPR spectra acquired by the POF-SPR sensor in buffer for the different steps of the surface functionalization process and for the first injection of serum (not spiked).

**Figure 7:**
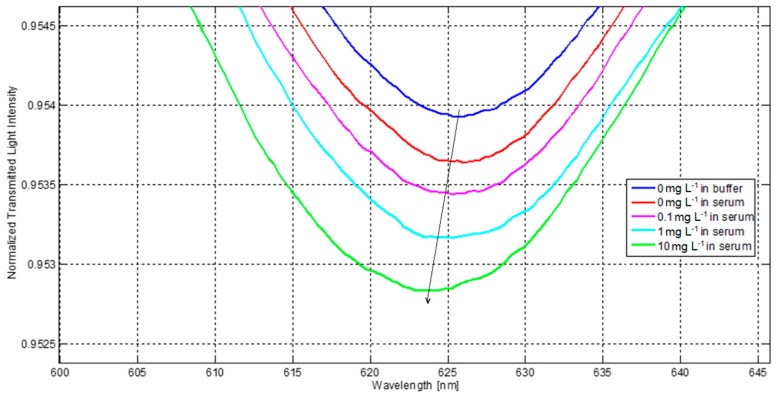
Normalized SPR spectra acquired by the POF-SPR biosensor for different analyte concentrations in serum.

The first SPR spectra were recorded in order to examine the influence of the sensor surface modification on the resonances. Figure 6 shows the SPR resonances obtained after each step of the functionalization process, as described in Section 2.4. The wavelength of the SPR resonance is shifted to lower values (blue-shift) after the receptor (IgG) is immobilized on the surface. The blocking of the surface with BSA did not lead to a change in resonance wavelength, demonstrating the good surface coverage. Moreover, the absence of a detectable wavelength shift after the first injection of human serum, which contains other kinds of IgG (but not the specific one), demonstrated the good specificity of the sensor that should result in a signal only in presence of anti-mouse IgG (specific analyte). The preliminary results on the assay in human serum are shown in Figure 7 that details the shift of the SPR resonance at around 626 nm, obtained for the different concentrations of analyte (from 0 to 10 mg/L) spiked inhuman serum. Even in this case, the resonance wavelength is shifted to lower values when the analyte concentration increases.

The total shift of the SPR resonance detailed in Figure 7 is roughly 2.2 nm, which is slightly lower than that obtained when the assay was performed in buffer for the same analyte concentration of 10 mg/L (2.7 nm from Figure 5). This could be ascribed to the higher complexity of the serum matrix with respect to simple PBS buffer, which implies a decrease of the antigen mobility [26].

These preliminary results represent a new paradigm for biochemical sensing applications. In fact, the new sensor system simplifies the use of the POF-SPR sensor platform and increases its reliability for biochemical sensing applications. The use of the integrated system could also allow the implementation of an assay protocol including a regeneration step to obtain the specific biolayer available for a new interaction in consecutive assay cycles. Several examples of regeneration procedures can be found in the literature [27] and represent a future issue, which will be taken into account with the exploitation of the proposed POF-SPR system to a real application.

The other kind of regeneration which could be considered in this type of biosensors, is the complete restoration of the gold sensor surface by removing not only the bound analyte but also the immobilized bioreceptor and the functional layer (thiols in most cases when using gold). This step is rarely performed in SPR biosensors because of the difficulty in removing the thiols from the gold surface [28]. This last kind of regeneration requires strong treatments, such as electrochemical cleaning [29], ultraviolet/ozone exposure [30], and ammonia-hydrogen peroxide mixture (APM) solution stripping [31], which are often performed out of the integrated fluidic system. Being the aim of this work the demonstration of the feasibility of the integration of a POF-SPR sensor into a thermo-stabilized flow cell, this kind of regeneration was beyond the scope of the work.

## 4. Conclusions

In this work, the first experimental measurements obtained with a POF-SPR sensor integrated with a thermo-stabilized flow cell have been presented. In particular, the effectiveness of the new complete optical sensing platform has been demonstrated thanks to the development of the microfluidic system, which has been used for both the functionalization of the sensor surface and the antibody-antigen binding experiments obtained in buffer solution (PBS) and in human serum. These experimental results show that the POF-SPR sensor platform is appropriate for biochemical applications based on fluidics, even in a real and complex environment such as serum.
